# Unimpaired vision is an indispensable prerequisite for safe driving

**DOI:** 10.4102/phcfm.v16i1.4810

**Published:** 2024-12-10

**Authors:** Josef Finsterer

**Affiliations:** 1 Department of Neurology, Faculty of Neurology, Neurology and Neurophysiology Center, Vienna, Austria

It was interesting to read the article by Tamenti et al. on a random-effects meta-analysis using STATA18 of 17 cross-sectional or observational studies on the association between visual impairment and road traffic accidents.^1^ They concluded that there is an association between road traffic accidents and visual function and that targeted interventions such as regular eye examinations of drivers at appropriate intervals are needed.^1^ The study is convincing, but some points should be discussed.

The first point is that visual function has only been associated with impairment of the eye.^1^ However, vision can also be affected by central nervous system (CNS) disorders, heart disease, ear nose throat (ENT) disorders, medications or illicit drugs, whether or not eye disease is present. Central nervous system disorders that may be associated with visual problems include cerebrovascular disease, encephalitis, epilepsy, migraine, posterior reversible encephalopathy syndrome, endocrine disease, stroke-like episodes in mitochondrial disease, neurodegenerative disease and optic nerve disease. Ear nose throat diseases that can be associated with visual impairment include pansinusitis, which also affects the sphenoid sinus and can be complicated by cavernous sinus syndrome. Cardiac diseases that can be associated with visual impairment include heart failure, arterial hypertension, atrial fibrillation and malignant cardiac arrhythmias. Endocrine diseases that lead to visual disturbances include Graves’ disease, hyperglycaemia or hyperparathyroidism (calcification of the optic nerve). Various medications can impair visual function, including non-steroidal anti-inflammatory drugs, glucocorticoids, neuroleptics, antidepressants (venlafaxine, paroxetine, fluvoxamine), antiarrhythmics or anti-allergic drugs.

The second point is that the connection between visual impairment and traffic accidents is obvious. In most countries, a driving licence is only issued after a successful eye test. If a person’s vision falls below a certain threshold, they are not allowed to drive a motor vehicle. The problem with these tests, however, is that they do not detect temporary or episodic visual impairments, which often occur unnoticed and can lead to an acute inability to drive. This is particularly the case with occipital seizures, amaurosis fugax, visual auras or acute heart failure syndromes. Such events endanger not only the driver but also other road users.

Overall, this interesting study has limitations that put the results and their interpretation into perspective. Addressing these limitations could strengthen the conclusions and reinforce the message of the study. Unimpaired vision is an essential prerequisite for safe driving and must be regularly monitored by the authorities.
